# Relationship of the nursing practice environment with the quality of care and patients’ safety in primary health care

**DOI:** 10.1186/s12912-023-01571-8

**Published:** 2023-11-02

**Authors:** Pedro Lucas, Élvio Jesus, Sofia Almeida, Beatriz Araújo

**Affiliations:** 1 Center for Interdisciplinary Research in Health, Instituto Ciências da Saúde, Universidade Católica Portuguesa, Rua de Diogo Botelho, Porto, 1327, 4169-005 Portugal; 2 Nursing Research, Innovation and Development Centre of Lisbon (CIDNUR), Escola Superior de Enfermagem de Lisboa, Av. Prof. Egas Moniz, Lisbon, 1600-096 Portugal

**Keywords:** Patient safety, Primary Health Care, Quality of nursing care, Work Environment

## Abstract

**Background:**

Nursing practice environment has impact on the quality of nursing care and on patients’ and nurses’ outcomes, namely better performances of these healthcare workers. Improving the nursing practice environment is a low-cost organizational strategy to achieve better patients’ outcomes and retain qualified nurses, thus improving nursing care of units, healthcare organizations and healthcare system. This study aims to analyse the relationship between nursing practice environment and the nurses` perception of quality of care, patient safety, and safety culture in Primary Health Care in Portugal.

**Methods:**

We conducted a descriptive, analytical, and cross-sectional study using data from RN4CAST Portugal. The sample was composed of 1059 nurses from 55 Health Center Groups of the mainland Portugal, 15 Health Centers of the Autonomous Region of Madeira and 6 Health Centers of the Autonomous Region of the Azores. Multivariate analysis and correlation analysis methods were used for data processing.

**Results:**

Nurses consider that, in the Portuguese Primary Health Care, there is a mixed and unfavourable nursing practice environment, with a perception of a good quality of care, and both acceptable patients’ safety and safety culture. The Collegial Nurse-Physician Relations and Nursing Foundations for Quality of Care dimensions to have the best ratings. The perception of Primary Health Care nurses on the dimension Nurse Participation in Organization Affairs was the one that showed the lowest score, followed by Staffing and Resource Adequacy and Nurse Manager Ability, Leadership, and Support of Nurses. Based on perception of nurses, the relationship between the nursing practice environment and the safety culture is higher, followed by the quality of care and patients’ safety.

**Conclusions:**

The perception of Primary Health Care nurses is that there is an unfavourable and mixed nursing practice environment, with good quality of care, and acceptable patient safety and safety culture. The quality of the nursing practice environments is associated to better quality and safety of care. Thus, improving the nursing practice environments in healthcare organizations is a low-cost organizational strategy to achieve greater patients and nurses’ outcomes, improving the quality of nursing care to patients in the Primary Health Care units.

## Background

Few studies have assessed the nursing practice environment in Portugal, and its implications. In the Primary Health Care context, studies are scarce [1–5]. Nursing in the Portuguese context is associated with high stress, low staffing levels in the workplace, and low recognition of skills and experiences [1]. The nursing practice environment should be a major focus of concern for Health Systems to avoid crises in two areas: shortage of nurses and patient safety [2].

The World Health Organization considers that the work environment is an important factor in the recruitment and retention of health professionals and that the characteristics of the work environment affect the quality of care, directly and indirectly [3]. In this respect, the World Health Organization has proposed a model for promoting healthy workplaces to promote the health of workers and improve physical and psychosocial environments [4]. Improving the work environment is a vital goal to ensure the effectiveness and develop the motivation of health human resources [5].

The International Council of Nurses considers that working environments should be positive and defines them as contexts that support excellence and the work of professionals in the right way [6]. They should ensure the personal health, safety and well-being of professionals, support the quality of care and improve the motivation, productivity and performance of individuals and organizations [6]. The interaction between nurses and the work environment is significant and implies that the effect of these professionals on the environment is conditioning and, alternately, the effect of the work environment conditions the nurses [7]. The complex social and professional environments where nurses develop their practices and where there are permanent needs of health workers are called professional practice environments [3]. The nursing practice environment consists of a set of specific or abstract characteristics of an organization, related to both the processes and structures of that organization, which are considered by nurses as facilitators or constraints of their professional practice [5, 8–13]. The nursing practice environment is the most influential factor with the greatest impact on nursing outcomes and on perceptions of quality of care and patient safety [12]. These issues are particularly important in the context of the global crisis that has affected countries in recent years [5, 12].

The nursing practice environment can affect the quality of nursing care and productivity [8, 14–18]. Regarding the safety of patients with nursing care, it depends mainly on the nurse staffing in the teams [19]. Compared to the nursing practice environment in hospital settings, knowledge and scientific evidence are scarce regarding the Primary Health Care settings [11, 12, 20–22], which affects the expansion of the profession in this context [11, 12, 22–24]. With this study, we intend to contribute to reducing this gap in scientific evidence, both in Portugal and around the world.

This study aims to analyse the relationship between of nursing practice environment and the nurses`perception of quality of care, patient safety, and safety culture in Primary Health Care.

## Methods

### Study design and setting

This study, within the scope of Primary Health Care, is part of a larger research project, which is the RN4Cast Portugal Project (2018), a replication of the international RN4Cast study [25, 26]. RN4CAST aimed to describe nursing practice environment in Portuguese healthcare organizations in terms of human and material resources, and other variables such as patient safety and quality of care. We conducted an descriptive, analytical and cross-sectional study aiming to analyse the relationship between nursing practice environment and the nurses` perception of quality of care, patient safety and safety culture in Primary Health Care in Portugal.

### Study population and eligibility criteria

The study sample consisted of all nurses with any type of contractual relationship with the Primary Health Care of the National Health Service working in the 55 Health Center Groups in mainland Portugal, 15 Health Centers in the Autonomous Region of Madeira and 6 Health Centers in the Autonomous Region of the Azores. The total sample consisted of 1059 nurses.

According to the Portuguese Board of Nurses, there were 7383 nurses working in primary health care. Since our sample consisted of 1,079 nurses, we obtained a 14.3% response rate. This study showed a wide national representation of nurses and, for the first time in Portugal, a territorial and numerical coverage in a Primary Health Care study. The territorial coverage due to the fact that nurses from all the Health Center Groups on the mainland territory and nurses from the two autonomous regions of the Azores and Madeira responded. The sample size is the largest observed in studies with Primary Health Care nurses in Portugal, to this moment.

### Instrument

As a data collection tool, we used the Nurse Survey Instrument (Core Nurse Survey) with the structure defined by the international RN4Cast consortium and consisting of four Sections [9, 18, 26]: Section A assesses the nursing practice environment measured by the PES-NWI scale and other questions measuring the characteristics of nurses’ work; Section B assesses questions about the quality and safety of care; Section C assesses the nurses’ work organization; and Section D on the sociodemographic characterization of the professionals. The PES-NWI scale by Lake (2002) [8], translated and validated by Amaral et al. [27], has 31 items, with responses on a 4-point Likert-type scale, has been used in several studies [9, 18, 25, 26, 28].

The questions on Quality of Care were given in response to the items in group B − 1, 2, 3 and 5 - of the Nurse Survey Instrument, on a Likert-type scale of 3 and 4 points [29]. The question on Patients’ Safety was given in response to item 4 of group B of the Nurse Survey Instrument, on a 5-point Likert-type scale [29]. This item was used in the studies of Aiken et al. [25], Alenius et al. [30], Coetzee et al. [18] and Kirwan et al. [31]. The questions on Safety Culture were given in response to the six items in group B6 of the Nurse Survey Instrument [29]. These items are part of a larger questionnaire which is the Hospital Survey on Patient Safety Culture from the Agency for Healthcare Research and Quality’s [30]. The data collection instrument was used with the structure that the international RN4Cast consortium defined and underwent translation and cross-cultural validation for Portugal [29].

### Data collection

This study used data from the RN4Cast Portugal Project (2018). This Project was developed by a research team from the Portuguese Catholic University, coordinated by the Professor Élvio Jesus and is a replication of the international study RN4Cast [25, 26] of which it is part. Data collection period took place between November 2017 and May 2018. The study was disclosed by the Portuguese Board of Nurses on its institutional website, at the request of Portuguese Catholic University, and nurses were asked to participate through a link to access the Nurse Survey Instrument. This instrument included an introductory note explaining the study and its objectives, as well as the guarantee of anonymity and confidentiality of the answers.

### Data analysis

We used the Statistical Package for the Social Science - IBM®-SPSS Statistics® version 26.0 statistical software for data processing and multivariate analysis and correlation analysis methods. The initial statistical analysis was an exploratory analysis of the variables to verify the assumptions for their use and the characterization and frequency of data. Categorical variables were described by absolute (n) and relative (%) frequencies; quantitative variables were described by mean, standard deviation, minimum and maximum. Correlations between quantitative variables were analysed by the Pearson’s correlation test. The significance level was 0.05, and two-sided hypotheses.

### Ethical issues

This study complies with the Declaration of Helsinki guidelines on an ethics committee opinion for approval [32]. The RN4Cast Portugal Project had a favourable opinion from the Ethics Committee, of the Regional Center of Porto, of the Portuguese Catholic University, no. 03/2018, from 14th May 2018. Nurses who answered the Nurse Survey Instrument questionnaire of the RN4Cast Project (2018) in the link provided, participated in a free and consented way, confirmed by submitting the online form. Informed consent was obtained from all study participants. The beginning of the online response form included an introductory note explaining the commitment to safeguarding the subjects’ anonymity and data confidentiality in addition to clarifying the study and its objectives.

## Results

### Sociodemographic and professional characteristics

The sample under study corresponds to 14.3% of all Portuguese primary health care nurses, with a mean age of 43.5 years (SD = 7.9 years), predominantly female (85.8%).

Most of the nurses had a degree in Nursing (98.9%), 1.1% had a bachelor’s degree (the lowest level in the Portuguese nursing education) and 54.7% were nurse specialists. The mean time of profession was 20.5 years (SD = 7.8 years), they had worked in the organization for 14.5 years (SD = 10 years) and in the unit where they provide care for 9.6 years (SD = 7.6 years). Primary Health Care are the sole job for 83.2% of nurses and 57.3% said that they were satisfied and very satisfied with their choice of Nursing as a career.

### Nursing practice environment

The dimension Nurse Participation in Organization Affairs (NPOA) was the one that showed the lowest score with $\overline{x}$=2.2 (SD = 0.5), followed by Staffing and Resource Adequacy (SRA), with $\overline{x}$=2.4 (SD = 0.6) and Nurse Manager Ability, Leadership, and Support of Nurses (NMALSN), with $\overline{x}$=2.4 (SD = 0.6) (Table 1). Nurses considered the Collegial Nurse-Physician Relations (CNPR) and Nursing Foundations for Quality of Care (NFQC) dimensions to have the best ratings, with $\overline{x}$=2.8 (SD = 0.6) and $\overline{x}$=2.9 (SD = 0.5), respectively. The average value of the global scale was $\overline{x}$=2.5 (SD = 0.4) which represent unfavourable nursing practice environment.

**Table 1:** Mean values of the PES-NWI scale dimensions in Primary Health Care in Portugal

PES-NWI scale dimensions	N	Mean	SD
Nurse Participation in Organization Affairs	1059	2.2	0.5
Nursing Foundations for Quality of Care		2.9	0.5
Nurse Manager Ability, Leadership, and Support of Nurses		2.4	0.6
Staffing and Resource Adequacy		2.4	0.6
Collegial Nurse–Physician Relations		2.8	0.6
PES-NWI TOTAL		2.5	0.4

With regard to Quality of Care (Table 2), nurses describe the quality of nursing care delivered to patients in their unit/service as good in 63.6% of the cases. If we consider the positive evaluation, we have 80.8% of the opinions. The mean value of item 1 was good ($\overline{x}$=3.0; SD = 0.7). It is remarkable the response of 17.2% of the nurses who considered that they had excellent quality of care. When rating their degree of confidence regarding their patients’ ability to manage their care after discharge, 76.3% are confident, and if we consider the positive evaluation, they total 84.1% of the opinions. The mean value of this item was very close to confident ($\overline{x}$=2.9; SD = 0.5).

In relation to their degree of confidence in the way the management of the organization will solve the problems related to patients care reported by them, 47.9% were confident. Only 2.6% were very confident, making a total of 50.9%. The average value for this item was between not very confident and confident ($\overline{x}$=2.4; SD = 0.7). The responses to this item contrast with the high response percentages to the other items. In the item about the quality of care provided to patients in their organization in the past year, 49.9% consider that it has remained the same, 30.1% that it has improved and 19.9% that it has deteriorated ($\overline{x}$=2.1; SD = 0.7). The overall mean value for quality of care was $\overline{x}$=2.7 (SD = 0.5) which represent good quality.

**Table 2:** Absolute and relative classifications and frequencies of Quality of Care items

	Classification	N	%
In general, how would you describe the quality of nursing care provided to patients in your unit/service?	Poor	21	2,0
	Reasonable	182	17,2
	Good	674	**63,6**
	Excellent	182	17,2
How confident are you in your patients’ ability to manage their care after discharge?	Nothing confident	11	1,0
	Unconfident	157	14,8
	Confident	808	**76,3**
	Very confident	83	7,8
How confident will the organization’s management resolve the patient care issues you report?	Nothing confident	97	9,2
	Unconfident	427	40,3
	Confident	507	**47,9**
	Very confident	28	2,6
It considers that in the last year the quality of care provided to patients in its organization …	Worse	211	19,9
	Remained the same	528	**49,9**
	Improved	319	30,1

About Patients’ Safety (Table 3), when nurses were asked about the general classification of their unit in terms of patient safety, 46.2% considered it acceptable, totalling 80.0% of positive answers in this item. There was no excellent response. The mean value for this item was acceptable ($\overline{x}$=3.2; SD = 0.9). The overall mean value for patients` safety was ($\overline{x}$=3.2; SD = 0.9) which represent acceptable value.

**Table 3:** Classification and absolute and relative frequency of Patient Safety item

	Classification	N	%
Please give an overall rating to your unit/service in terms of patient safety.	Flawed	91	8,6
	Poor	88	8,3
	Acceptable	489	**46,2**
	Very good	358	33,8
	Excellent	0	0

Regarding the Safety Culture (Table 4) and with regard to the question whether they feel that their mistakes are used against them, 38.2% of the nurses replied that they agree and totally agree, compared to 31.6% who said they disagree and totally disagree. The mean value of this item was in Neither Agree/Not Disagree ($\overline{x}$=2.9; SD = 1.1). When asked about the aspects that are neglected during the patients’ transference, the nurses’ answers in agreement were 37.6%, against 34.2% in disagreement. The mean value of this item was Neither Agree/Not Disagree ($\overline{x}$=2.9; SD = 1.1).

Regarding the question on freedom to question the decisions or actions of their superiors, the nurses’ response in Strongly Agree and Agree was 37.3%, and the responses in Strongly Disagree and Disagree were 35.7%. The mean value of this item was very close to Neither Agree/Not Disagree ($\overline{x}$=3.0; SD = 1.1). Most nurses (70.4%) totally agree and agree that in their unit they discuss ways to prevent errors from being repeated. However, 16.4% negatively replied totally disagree and disagree. The mean value of this item was closer to disagree ($\overline{x}$=3.6; SD = 1.0). Regarding receiving feedback on changes implemented from event reports, 41.8% of the sample responded positively Agree totally and Agree, against 30.3% of negative responses (Disagree totally and Disagree). The average value for this item was Neither Agree/Not Disagree ($\overline{x}$=3.1; SD = 1.1). The overall mean value for safety culture was $\overline{x}$=3.1 (SD = 0.7), which represent acceptable value.

**Table 4:** Classification and absolute and relative frequency of Safety Culture items

	Classification	N	%
Professionals feel that their mistakes are used against them.	I totally disagree	69	6,5
	Disagree	266	25,1
	I neither agree nor disagree	317	29,9
	Agree	269	25,4
	I totally agree	136	12,8
	Omitted	2	0,2
Some aspects are neglected during the transfer of patients between services.	I totally disagree	52	4,9
	Disagree	310	29,3
	I neither agree nor disagree	294	27,8
	Agree	294	27,8
	I totally agree	104	9,8
	Omitted	5	0,5
Professionals feel free to question the decisions or actions of their superiors.	I totally disagree	90	8,5
	Disagree	288	27,2
	I neither agree nor disagree	284	26,8
	Agree	354	33,4
	I totally agree	41	3,9
	Omitted	2	0,2
In this service we discuss ways to prevent mistakes from being repeated.	I totally disagree	50	4,7
	Disagree	124	11,7
	I neither agree nor disagree	135	12,7
	Agree	595	56,2
	I totally agree	150	14,2
	Omitted	5	0,5
We received *feedback* on the changes implemented from event reports.	I totally disagree	90	8,5
	Disagree	231	21,8
	I neither agree nor disagree	292	27,6
	Agree	400	37,8
	I totally agree	42	4,0
	Omitted	4	0,4
The interventions at the organizational management level demonstrate that customer safety is a top priority.	I totally disagree	92	8,7
	Disagree	243	22,9
	I neither agree nor disagree	302	28,5
	Agree	352	33,2
	I totally agree	68	6,4
	Omitted	2	0,2

### Relationship between nursing practice environment and the nurses` perception of quality of care, patient safety and safety culture

The higher the nursing practice environment value in Primary Health Care, the higher were the Safety Culture scores with a moderate correlation association (r = 0.64), followed by Quality of Care with a moderate association (r = 0.61), and Patients’ Safety with a moderate association (r = 0.47) (Table 5).

With regard to the nursing practice environment dimensions, this study found that the higher the value of: NPOA, the higher is the Safety Culture rating, with a moderate correlation association (r = 0.56), followed by the Quality of Care with a moderate association (r = 0.48); NMALSN, the higher is the Safety Culture rating, with a moderate correlation association (r = 0.48), followed by the Quality of Care with a moderate association (r = 0.41); NFQC, the higher is the Quality of Care rating, with a moderate correlation association (r = 0.54), followed by Safety Culture with a moderate association (r = 0.51); SRA, higher is the rating of Quality of Care with a weak association (r = 0.39), followed by Safety Culture with a weak association (r = 0.34); and of NPRC, higher is the rating of Safety Culture with a moderate correlation association (r = 0.43), followed by Quality of Care with a moderate association (r = 0.41).

The higher the Patients’ Safety score, the higher the Quality of Care score, with a strong correlation (r = 0.79), followed by Safety Culture with a moderate association (r = 0.50). The higher the Quality of Care score, the higher the Patients’ Safety score with a strong association (r = 0.79), followed by Safety Culture, with a moderate association (r = 0.64). The higher the Safety Culture score, the higher the Quality of Care score, with a moderate correlation (r = 0.64), followed by Patients’ Safety with a moderate association (r = 0.50). When analysing the association of the nursing practice environment, NPOA, NFQC, NMALSN, SRA and CNPR dimensions, we find a weaker association with Patients’ Safety. Patients’ Safety shows weak associations with NPOA, NMALSN and SRA, with r values between 0.30 and 0.35. The SRA dimension is the one which shows low correlations with Quality of Care, Safety Culture and Patients’ Safety, with r values between 0.30 and 0.39.

As regards nursing practice environment, between the dimensions themselves, this study found the weakest association between CNPR and NMALSN with r = 0.27 and the strongest association, moderate, between NPOA and NMALSN with r = 0.63. The nursing practice environment dimensions are positively correlated with each other with the highest value between NMALSN and NPOA with 0.63, followed by NFQC with 0.46. The lowest correlation value is observed between NMALSN and CNPR with 0.27, followed by SRA with 0.30.

**Table 5:** Correlations of variables

	NMALSN	NFQC	SRA	CNPR	NPE	Patient Safety	Quality Care	Safety Culture
NPOA	0.63**	0.46**	0.43**	0.41**	0.90**	0.35**	0.48**	0.56**
NMALSN		0.39**	0.30**	0.27**	0.74**	0.34**	0.41**	0.48**
NFQC			0.33**	0.45**	0.70**	0.42**	0.54**	0.51**
SRA				0.32**	0.62**	0.30**	0.39**	0.34**
CNPR					0.59**	0.36**	0.41**	0.43**
NPE						0.47**	0.61**	0.64**
Patient Safety							0.79**	0.50**
Quality Care								0.64**

** p < 0.01

## Discussion

From the findings of this study, we can state that the nurses who participated in this study consider there is an unfavourable and mixed nursing practice environment in Primary Health Care in Portugal, with a perception of good quality of care, and acceptable patient safety and an acceptable safety culture.

The classification of unfavourable nursing practice environment followed the proposal of Lake [8] and Zangaro & Jones [33]. If we apply the Lake & Friese’s (2006) classification [34] we verify that the nursing practice environment in Primary Health Care in Portugal is of the mixed type, since we have two dimensions (NFQC and CNPR) with average values above 2.5. The study of Jesus et al. [35] obtained a rating of an unfavourable nursing practice environment, which is in line with the results of this study, as in You et al. [28]. Regarding the classification of mixed, in this study and in that of Jesus et al. [35] in Portugal, the nursing practice environment is mixed, as in the study of Rabie et al. [23], in South Africa, and in Spain [21]. The other studies in Primary Health Care in Spain, by Gea-Caballero et al. [36] and by Parro-Moreno et al. [21] showed favourable nursing practice environment, as in the study in Shanghai, China, by Wang et al. [37] and in that of Jarrín et al. [38] in California, USA. In the study by Nantsupawat et al. [39] the Thai hospitals where there is a favourable nursing practice environment are the university hospitals that are subject to accreditation for Quality. The remaining hospitals without Accreditation Systems, the nursing practice environment are unfavourable [39]. Nurses from hospitals in South Africa also assessed their nursing practice environment as unfavourable in the study by Coetzee et al. [18].

Our findings show that the better nursing practice environment, the higher Quality of Care, higher Patients’ Safety and higher Safety Culture, which is in line with those of Nantsupawat et al. [39].

With regard to the relationship between nursing practice environment dimensions, the highest occurred between NPOA and NMALSN, followed by NPOA and NFQC, as in Klopper et al. [9], and NFQC and CNPR. In the study by De Pedro-Gomez et al. [40], the highest association occurred between NPOA and NFQC.

The relationship of nursing practice environment is greater with Safety Culture, followed by Quality of Care. Also, in the study of Abraham et al. [41], a favourable nursing practice environment is associated with high Quality of Care. In the study of Kirwan et al. [31], a favourable nursing practice environment is associated with a high Safety Culture.

As the nursing practice environment is one of the variables with the greatest influence on the quality of nursing care and Safety Culture, the fact that the Primary Health Care in Portugal are classified as unfavourable and mixed, are aspects to be considered for a continuous improvement of the nursing practice environment at this level of care, with relevant information for nurse managers and executive directors of the Health Center Groups and for health policy makers. It is important to improve nursing practice environment to provide these health organizations with conditions that promote favourable nursing practice environment, with satisfied patients and professionals who feel and see in their practices a better quality of nursing care with positive results for both patients and nurses.

The nurses’ perception is that they are satisfied with the delivery of care focused on its quality and with the multidisciplinary relationships. They considered the dimensions related to the management support and performance, and the exercise of leadership and participation in the governance of the organization as unfavourable. The adequacy of resources is also unfavourable, conveying a worrying reality of staffing levels with major impacts on patients’ and nurses’ outcomes.

With regard to Quality of Care, 19.2% of nurses reported the quality of nursing care delivery as poor or reasonable, identical to the studies of Coetzee et al. [18], Aiken et al. [16] and Aiken et al. [25] (in England, Finland, Norway and the USA), and below the 29.0% in the study by You et al. [28] and the 32.0–35.0% in Germany, the Netherlands and Spain in Aiken et al. [25]. This result is in line with the previous RN4Cast Portugal study by Roque [29], as well as the 63.6% good response value. The nurses who described the quality of care as excellent were 17.2%, higher than the 13.6% of nurses in hospital settings in the study of Roque [29].

In the answer about not being confident that their patients can take care of themselves after discharge, it was 15.8% of the nurses, a value much lower than all nurses from the 12 European countries and the USA who had answers between 28.0% and 74.0% in the studies of Aiken et al. [25], Aiken et al. [16] (with answers between 31.7% and 73.2% in Scotland, England, the states of Columbia and Pennsylvania) and in Coetzee et al. [18] with 32.7%. In this item, we obtained about 84.1% of the nurses who are confident, a value higher than the 71.4% in the study of Roque [29]. About half of the nurses, 49.5%, are not confident that the organization’s management will solve problems related to patient care, as in the study of Roque [29], a value higher than the 44.9% in Coetzee et al. [18] and the 45.7% in You et al. [28] and lower than the nurses from the 12 European countries and the USA who had responses between 57.0% and 87.0% in Aiken et al. [25].

About the quality of care provided by the unit in the last year, 19.9% of the nurses considered that it had worsened. When associated with the health region, this study found that nurses from the region of Alentejo had the highest scores in this dimension of quality of care and nurses from the region of Algarve had the lowest score in this dimension.

This study found that the Quality of Care has a higher association with Patients’ Safety, Safety Culture and the nursing practice environment, according to the nurses’ perception. Nurses of the Primary Health Care in Portugal who participated in this study consider that there is a good quality of care, which is in general agreement with the results of the study of Coetzee et al. [18] in hospitals in South Africa. Nurses in Primary Health Care in Nigeria consider having an average quality in their services [42]. Underdeveloped countries may have lower quality scores due to less capacity to invest in Primary Health Care.

This study found that the nurses who participated in this study consider that there is a good quality of care in Primary Health Care in Portugal, which is partly due to the great effort that these professionals make. Regarding the previous study RN4Cast, in Jesus et al. [35], this study found that the perception of the quality of care is higher in Primary Health Care than that reported by nurses in hospital settings.

Regarding Patients’ Safety, 80.0% of the nurses gave a positive rating to the safety of patients in their unit. However, no nurse rated the care provided in their unit as excellent, which is consistent with the results in the remaining variables. In relation to the classification of poor and with failures, 16.9% of the nurses responded, a value like Sweden and below that of Greece and Poland, and twice as high as the classification of nurses from Belgium, England, Finland, Germany, Ireland, the Netherlands, Norway, Spain, Switzerland and the USA, which had one in every five nurses [25]. Patients’ Safety has higher association with the Quality of Care, followed by the Safety Culture and nursing practice environment, just as the study of Klopper et al. [9].

Nurses who participated in our study consider that patients’ safety is acceptable, better than hospitals in Greece, Poland, and Sweden, in Aiken et al. [25] with nurses rating patient safety as poor, as well as hospitals in South Africa in Coetzee et al. [18] study. Both studies derived from the RN4Cast international. Compared to the study of Kirwan et al. [31], nurses in Ireland consider patient safety to be very good or excellent. These authors refer that patient safety increases when there is a greater number of nurses, with favourable nursing practice environment and a higher level of training of these professionals [31], also following the observations of Aiken et al. [25], Bruyneel et al. [43], Kirwan et al. [31], Titlestad et al. [44] and You et al. [28]. With reference to the previous RN4Cast study in Portugal in hospital settings, this study found that the perception of Patients’ Safety was acceptable [29], a similar situation occurred in this study in Primary Health Care.

Relating to the Safety Culture, this study found that 38.2% of the nurses reported that they feel their errors are used against them and that there are aspects neglected when transferring patients (37.6%). In the USA hospital settings, in the study of Olds et al. [45], the responses regarding errors were 33.8% and higher regarding the aspects neglected when transferring patients (44.5%). Another study conducted in the USA in a hospital setting reported 41% regarding errors and 36% regarding aspects neglected during the transfer of patients [46]. The first study showed more variability than the second one regarding these results in Primary Health Care. On whether or not they have freedom to question the decisions or actions of their superiors, it was 35.7%, lower than the 44.5% of the study of Olds et al. [45] and the 38% of Carthon et al. [46]. When asked whether they discuss ways to prevent errors from recurring, 16.5% of nurses of this study responded that they agree, and 30.3% received feedback on changes implemented from event reports and on the organization’s management interventions, demonstrating that patient safety should be a top priority. In Olds et al. [45] they had 77.1% and 57.5% respectively, and in Carthon et al. (2019) [46] they had 87% and 73% respectively.

The nurses who participated in this study refer that the organization is not dynamic and interventive in strategies to promote patient safety, and it was found that Safety Culture has a greater association with nursing practice environment, Quality of Care and Patients’ Safety.

Nurses of Primary Health Care in Portugal who participated in our study consider there is an acceptable Safety Culture, which is in line with the findings of You et al. [28]. Nurses have more confidence in their ability to provide care that promotes patient safety. The fact that nurses feel that their mistakes are used against them is negative for the professionals’ engagement and organizational commitment and may even strongly influence the nursing practice environment in the teams. This is where the importance and maturity of the nurse manager’s intervention comes into play in understanding this malfunctioning in the units and changing nurses’ opinions.

Concerning the previous RN4Cast study in Portugal, in hospital settings, this study found that the perception about the Safety Culture was acceptable [29], as observed in this study in Primary Health Care. The scientific evidence is clear on the investment in training and the relationship between more training, better nursing practice environment, better quality and safety of care, and empowerment of human resources [25, 28, 31, 43, 44].

### Limitations and recommendations

This study shares limitations like other studies in health services based on cross-sectional data, such as the association observed in the sample not reflecting what occurs in the population and limiting the inference of causality as well as the collection of data by self-report.

Although we had a sample with a national representation and a major territorial coverage in Primary Health Care for the first time in Portugal, including the Autonomous Regions of Madeira and Azores, the modest response rate of 14.3% may be a limitation of the study, associated with the fact that data collection was conducted online. Currently, and due to the impacts of the pandemic caused by COVID-19, data collection through questionnaires has become more widespread through digital means, which may lead to a lack of interest in nurses to answer.

In the future, a longitudinal study may be designed, as well as the inclusion of other variables related to nursing practice environment, such as staffing, workload, information systems, nurses’ well-being, and establish causal links between nursing practice environment, which are intended to be favourable, and better outcomes for patients, nurses, and healthcare organizations. The considerable dimension of the Health Center Groups and the consequent number of nurses are a facilitator to access data sets that allow for enriching conclusions for the contexts of nursing practices, nursing management and the efficiency of organizations.

Furthermore, the nature of the relationships between the study variables transcends any recent or short-term trends in healthcare; otherwise, the issue under study would no longer be a problem and would have been solved long ago. We faced the difficulty of finding few studies in Primary Health Care context on nursing practice environment and none on RN4Cast study in Primary Health Care in other countries.

The conditioning factors of other health professional groups may be studied in the future, as well as the patients’ opinions about the care provided. In this way, it will be possible to compare with various studies and with this type of results on the nursing practice environment.

## Conclusions

The perception of Primary Health Care nurses is that there is an unfavourable and mixed nursing practice environment, with good quality of care, and acceptable patient safety and safety culture. This study found a perception of good quality of care in Primary Health Care in Portugal, which is partly due to the great effort that nurses make, despite having a mixed and unfavourable nursing practice environment This study found that the strong association between quality of care, safety culture, patient safety and adequacy of human and material resources is a relationship that confirms findings in other scientific evidence and in other care settings.

It is important to continue to study the nursing practice environment in organizations so as to contribute to the reorientation of nurses’ and nurse managers’ practices and for health policies to benefit from scientific evidence for their decision-making, with regard to the promotion of conditions that foster favourable nursing practice environment in organizations.

The study has generated the necessary scientific basis that may support a set of decision-making in health policies and the development of efficient strategies for nurse managers. Improving nursing practice environment in healthcare organizations is a low-cost organizational strategy to achieve greater patients’ outcomes and to retain qualified nurses, improving the quality of nursing care for units, organizations, and the healthcare system.

## Data Availability

All relevant data are included with in the manuscript document.

## List of abbreviations

CNPR: Collegial Nurse–Physician Relations
NFQC: Nursing Foundations for Quality of Care
NMALSN: Nurse Manager Ability, Leadership, and Support of Nurses
NPE: Nursing Practice Environment
NPOA: Nurse Participation in Organization Affairs
SRA: Staffing and Resource Adequacy
